# Removal of the Antibiotic Amoxicillin from Polluted Freshwater by Biosorption Using the Living Biomass of the Microalga *Chlamydomonas reinhardtii*

**DOI:** 10.3390/toxics13070520

**Published:** 2025-06-21

**Authors:** David Fernández, Julio Abalde, Enrique Torres

**Affiliations:** Laboratorio de Microbiología, Facultad de Ciencias, Universidade da Coruña, Campus de A Zapateira, 15071 A Coruña, Spain; davidfdz182@gmail.com (D.F.); abaldej@udc.es (J.A.)

**Keywords:** microalga, amoxicillin, biosorption, antibiotic, bioremediation, living biomass

## Abstract

The environment is undergoing a constant incorporation of new pollutants, which must be eliminated to avoid toxicity problems. Amoxicillin (AMX) is a widely used antibiotic today, and for this reason, it reaches natural media with the consequent environmental risk. Biosorption is an effective and environmentally friendly solution which can be used for the removal of AMX. In the present study, the properties of the living biomass of the microalga *Chlamydomonas reinhardtii* were studied to determine the capacity of this biomass to remove AMX. This biomass has demonstrated to have good qualities to remove AMX with a maximum capacity of 12.72 ± 0.57 mg g^−1^. Light was an important factor in increasing the removal capacity of this living biomass by 35.2%. Although this antibiotic underwent spontaneous degradation (unaffected by light), the presence of the biomass increased the amount removed and the removal rate. The amount removed by this biomass in the presence of light was always higher than the amount lost by spontaneous degradation. The kinetics that best adjusted was pseudo-second order. Maximum removal was obtained at pH 6. A point of zero charge and Fourier transform infrared spectrometry were used to characterize the biomass and study the process.

## 1. Introduction

Currently, antibiotics are widely used in a variety of fields, including medicine, veterinary medicine, agriculture, aquaculture, animal husbandry and beekeeping [1]. In fact, it is estimated that the use of antibiotics exceeds 100,000 tons per year [2]. In 2023, the World Health Organization (WHO) already published a list of 592 essential medicines for human health, 41 of which were antibiotics [3]. As a consequence of this use, many antibiotics reach aqueous effluents, generating a serious pollution problem [4]. After ingestion of an antibiotic, metabolites or even unmodified molecules of the antibiotic itself are produced and excreted with the urine and feces [5], constituting one of the main sources of contamination. In fact, antibiotics have been detected in drinking water, wastewater, aquifers and surface water [6]. The concentrations at which these compounds are generally found in water range from nanograms to micrograms per liter [7,8].

Antibiotics dispersed in natural environments can have important consequences for human health and ecosystems [2]. Their presence in water bodies is associated with toxicological problems due to their biomagnification capacity [9] (which indirectly contributes to the transport of pollutants in the food chain), selection of resistant bacterial strains (including potentially pathogenic bacteria) [9,10,11,12] and toxicity on organisms, which influences ecological processes [13], leading to changes in the structure and genetics of microbial communities [14].

The penicillin group is one of the most widely used groups of antibiotics, accounting for 70% of the antibiotics consumed in several countries [15]. Within this group is amoxicillin (AMX). AMX is a generic antibiotic [16] that is active against a broad spectrum of Gram-negative and Gram-positive bacteria, hence its wide use, and therefore, greater environmental risk. In fact, AMX is of concern because of the risk to the aquatic environment and human health [16]. Over 80% of AMX is excreted through urine [16], and it is one of the most widely reported antibiotics in wastewater [17]. It has been shown that wastewater treatment plants do not have the potential to degrade AMX completely, and residual concentrations of AMX can be found in drinking water sources [16]. This is the main reason why several techniques have been studied to remove this antibiotic from water bodies, such as the use of iron particles [18], NH_4_Cl induced activated carbon [19], UV and UV/H_2_O_2_ [20]. However, techniques considered more natural such as biosorption are preferred to remove pollutants from the natural environment [21], even when pollutants are at low concentrations [22], because these techniques do not produce secondary pollutants [23]. Moreover, biosorption is widely used given its low cost, simplicity, low carbon footprint and is considered as an environmentally friendly technique [24].

The main biological element of biosorption is biomass, where biological materials of different nature, from waste to microorganisms (such as bacteria, microalgae and fungi), can be used as sorbents [25]. Microalgal biomass is particularly attractive as biosorbent because the microalgal cells have a large amount of biopolymers such as cellulose, hemicellulose and cell wall proteins [26] that are important in the biosorption processes [27] because these molecules interact with pollutants, influencing the efficiency of the process [28].

*Chlamydomonas reinhardtii*, is a unicellular green microalga with mixotrophic behavior and a short life cycle [29]. This microalga is mainly found in freshwater and soils. It is widely used in environmental studies because it is easy to cultivate in laboratories and is a robust organism [30]. In addition, the bioremediation capacity of *C. reinhardtii* against xenobiotics such as drugs or chemicals is well known [31]. For this reason, the main objective of the present work was to determine the capacity of the living biomass of the microalga *C. reinhardtii* for the removal of AMX from freshwater. For this purpose, the kinetics of the process, the equilibrium isotherms, the effect of light and the influence of pH changes in the medium were determined. Few studies in biosorption use living biomass; however, the use of living biomass has the advantage of preserving the metabolic characteristics of the biomass. This means that AMX could also be removed by bioaccumulation, or could be metabolically degraded, and not only removed by bioadsorption as it would be using dead biomass.

## 2. Materials and Methods

### 2.1. Culture of the Microalga

*Chlamydomonas reinhardtii* was grown in 1 L glass bottles (Pyrex, Sigma-Aldrich, St. Louis, MO, USA) placed in a culture chamber at a temperature of 18 ± 2 °C with a light intensity of 68 µE m^−2^ s^−1^ and 12/12 h light/dark cycles. Deionized water enriched with GOLD-FWS medium was used to prepare the culture medium, which was sterilized at 121 °C for 20 min. The culture was blown with filtered air (0.22 µm pore size) at a flow rate of 10 L min^−1^. The microalgal biomass used in the experiments was obtained from this stock culture, during the exponential growth phase.

### 2.2. Amoxicillin Stock

The antibiotic tested in these experiments was amoxicillin (AMX). It is a β-lactam antibiotic belonging to the aminopenicillin group, and its chemical structure is C_16_H_19_N_3_O_5_S. The stock solution of the antibiotic was prepared using deionized water at a final concentration of 1 g L^−1^.

### 2.3. Reagents

The reagents used in this work were potassium (III) hexacyanoferrate (CAS = 13746-66-2; CIS = 237-323-3) (10 mM); ammonium hydroxide (CAS = 1336-21-6; CIS = 215-647-6) (50 mM); N-N dimethyl-P-phenyldiamine (DMPD) (CAS = 99-98-9; CIS = 263-723-2) (5 mM); sodium hydroxide (CAS = 1310-73-2; CIS = 215-185-5) (0.1 M) and hydrochloric acid (CAS= 7647-01-0; CIS = 231-595-7) (0.1 M). All these reagents and AMX were obtained from Sigma-Aldrich (St. Louis, MO, USA).

### 2.4. Biosorption Assays

Biosorption experiments were performed in 50 mL Kimax tubes (Sigma-Aldrich, St. Louis, MO, USA) using different concentrations of AMX. All assays were performed using a living biomass concentration of 1.1 g L^−1^ expressed as dry weight. To determine the volume that had to be taken from the microalgae stock culture to obtain this biomass concentration, the cell density of the stock culture was determined using a Neubauer chamber (Sigma-Aldrich, St. Louis, MO, USA). This value, together with the dry weight of the microalga, was used to calculate the volume that had to be taken from the microalgae stock culture to obtain the equivalent amount of dry biomass. This volume was centrifuged at 1700× *g* × 3 min to obtain the microalgal biomass which was resuspended in a known volume of sterile deionized water and transferred to the Kimax tubes. To these tubes, a volume of AMX stock solution was added to obtain the antibiotic concentrations used in the experiments: 0, 5, 10, 15, 20, 25, 50, 75 and 100 mg L^−1^. Finally, sterile deionized water with a pH = 7 was added to obtain a final volume of 50 mL. The tubes were incubated in the culture chamber at 18 ± 2 °C in an orbital shaker with shaking at 200 rpm and light intensity of 68 µE m^−2^ s^−1^.

For all experiments, samples were taken on days 0, 1, 4, 5, 6, 7, 8, 11, 12, 13 and 14. The samples were centrifuged at 6000× *g* × 2 min, the supernatant was obtained and stored in a freezer at −20 °C until AMX measurement. Moreover, the amount of biomass present in these samples was verified by counting the microalgal cells under the microscope using a Neubauer chamber. The cell density value obtained was converted into its dry weight equivalent in order to determine the amount of AMX removed per unit of biomass.

#### 2.4.1. Stability of AMX

To study the stability of AMX under the experimental conditions, and considering a possible photodegradation effect due to light on the antibiotic, experiments similar to the biosorption assays but without microalgal biomass were performed under both light and dark conditions (in the case of darkness, the tubes were covered with aluminum foil) at concentrations of 5, 10, 15, 20, 25, 50, 75 and 100 mg L^−1^ of AMX. Aliquots were extracted on days 0, 1, 4, 5, 6, 7, 8, 11, 12, 13 and 14 to determine the antibiotic concentration.

#### 2.4.2. Determination of the Effect of pH on Biosorption Capacity

To quantify the effect of pH on the biosorption capacity of AMX by the living biomass of the microalga *C. reinhardtii*, deionized water solutions with different pH values were used. These solutions were adjusted with NaOH or HCl as required. The pH values tested were 4, 6, 7 (pH at which the biosorption experiments were performed), 8 and 10. The procedure carried out was analogous to the biosorption tests, using AMX concentrations of 10, 50 and 100 mg L^−1^. To determine the stability of AMX at these pHs, controls were used under the same conditions as the biosorption experiments but with the absence of biomass. Aliquots were extracted on days 0, 1, 4, 5, 6, 7, 8, 11, 12, 13 and 14 and were treated in the same way as in the biosorption experiments.

### 2.5. Analytical Methods

#### 2.5.1. Determination of AMX Concentration

The determination of AMX concentration in the assays was based on a spectrophotometric method [32]. The method consisted of taking a 0.4 mL aliquot of the sample to which 0.2 mL of Potassium Hexacyanoferrate (III), 0.125 mL of Ammonium Hydroxide and 0.4 mL of DMPD were added. It was left to react for at least five minutes, and the absorbance was measured in a spectrophotometer (PharmaSpec UV-1700 UV/Vis, Shimadzu, Kyoto, Japan) at a wavelength of 660 nm. To relate absorbance to antibiotic concentration, a calibration curve was performed with different antibiotic concentrations in deionized water and processed in the same way.

#### 2.5.2. Determination of the Amount of AMX Removed

The amount of antibiotic removed per unit of biomass and the percentage removed were determined by the following:

(1) $$q_{t}=\frac{\left(C_{c}-C_{t}\right)\times V}{m}$$

(2) $$P_{t}=\frac{\left(C_{c}-C_{t}\right)\times 100}{C_{i}}$$

where *q_t_* (mg g^−1^) is the amount of antibiotic removed per unit of biomass at time *t* (d), *V* is the volume of the solution (L), *m* is the mass of the sorbent (g), *C_c_* is the AMX concentration in the control tubes (mg L^−1^) at time *t* and *C_t_* is the AMX concentration (mg L^−1^) of the samples with microalgal biomass at time *t*. *P_t_* is the percentage of AMX removed at time *t*, and *C_i_* is the initial AMX concentration (mg L^−1^).

#### 2.5.3. Analysis of Kinetics and Equilibrium Isotherms

The kinetics of the removal process over time and the isotherms at equilibrium were determined in order to obtain the properties of the living biomass of *C. reinhardtii* as an AMX biosorbent. For this purpose, the obtained data were fitted to different kinetic and isotherm models (Table 1).

### 2.6. Determination of the Biomass Zero Charge Point

The zero charge point (pH_zcp_) determines the pH value at which the biomass charge is zero. To determine the pH_zcp_, an amount of microalgal biomass of 1.1 g L^−1^ was used. This biomass was previously obtained by centrifugation at 6000× *g* × 2 min. This biomass was resuspended in 0.125 M NaCl solutions with different pHs from 2–13 (pH_i_). After 4 h of contact, the solutions were centrifuged at 6000× *g* × 2 min to obtain the supernatant, and the final pH of these supernatants was measured (pH_f_). The pH_zcp_ was obtained from a plot of ΔpH (pH_f_–pH_i_) against pH_i_. All pH measurements were made on a pH meter Basic 20 (Crison, Hospitalet de Llobregat, Barcelona, Spain).

### 2.7. Fourier Transform Infrared Spectroscopy (FTIR)

FTIR was used to determine the different functional groups present in the microalgal biomass that may be involved in AMX sorption. FTIR spectra were obtained on a FTIR spectrometer (Thermo Scientifc Nicolet iS10 with a module Smart iTX, Thermo Fisher Scientific, Waltham, MA, USA) by applying attenuated total reflection (ATR) within a range of 500–4000 cm^−1^ and a resolution of 4 cm^−1^. FTIR spectra were obtained for biomass after 14 days of AMX exposure and for biomass without antibiotic exposure. The biomass was obtained by centrifugation at 6000× *g* × 2 min and then dried in an oven at 50 ± 2 °C for 48 h prior to obtaining the spectra.

### 2.8. Statistical Analysis

All assays were conducted in triplicate. Data represent the mean of the three replicates ± SD. Non-linear regression was used to perform the fits to the kinetic and isotherm models. The coefficient *r_adj_*^2^ was used as a measure of goodness of fit. As necessary, a *t*-test, a one-way ANOVA, a two-way ANOVA or a multifactorial ANOVA was used to compare whether there were significant differences between groups of experimental data. When these differences were significant, a Tukey’s test were used to determine which groups had the differences. A significance level of *α* = 0.05 was set. It was verified that the requirements of each of the tests were met. SPSS software version 29.0.1.0 was used for these analyses.

## 3. Results

### 3.1. Study of AMX Stability Under Experimental Conditions

Control experiments were performed in the absence of microalgal biomass to determine the stability of the AMX antibiotic in freshwater both in the darkness and under light exposure. After 14 days of testing, a decrease in AMX concentration was observed in both illuminated and in those that were in darkness, and at all AMX concentrations tested. These results were analyzed using a two-way ANOVA (factors: AMX concentration at initial and final times, and initial tested concentration of the antibiotic). This analysis indicated that there were significant differences for the antibiotic concentration at initial and final times, both in the presence of light (*F*_1,32_ = 2967.91; *p* < 0.001) and in the darkness (*F*_1,32_ = 2867.43; *p* < 0.001), and also for the initial AMX concentration in light (*F*_7,32_ = 288.44; *p* < 0.001) and in darkness (*F*_7,32_ = 283.13; *p* < 0.001) at all tested antibiotic concentrations. That is, there was a significant loss of AMX during the assays without the presence of biomass. Moreover, this decrease was dependent on the initial AMX concentration. To determine whether light contributed to this loss, the data were analyzed using a *t*-test (comparing the concentration of AMX in the illuminated tubes with that in the tubes that were in darkness at 14 days). The result indicated that there was no significant difference between the results obtained in the presence of light and in the darkness (*t*_46_ = 0.03; *p* = 0.98). Therefore, there was a loss of stability of AMX which resulted in a gradual and spontaneous reduction in the initial antibiotic concentrations during the 14 days of testing, where photodegradation did not contribute to this reduction. During this period, approximately 14% of the initial AMX concentration disappeared at all AMX concentrations tested because of abiotic factors.

### 3.2. Effect of Microalgal Biomass on AMX Removal in Light and Darkness

**Figure 1:**
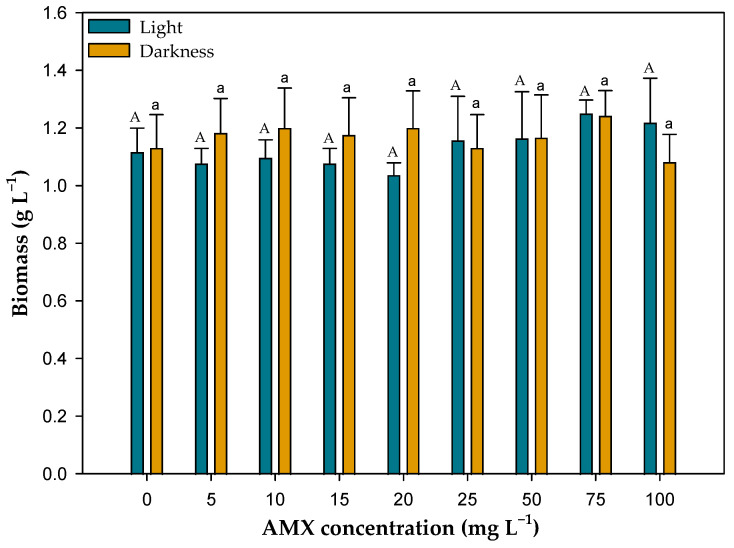
Final biomass of the cultures of *C. reinhardtii* exposed to different concentrations of AMX in both light and darkness. Different letters denote significant differences (*p* < 0.05) between the different initial concentrations of AMX in light (upper case letters) and in darkness (lower case letters).

Figure 1 shows the final biomass obtained at the end of the test time (14 days) in all the cultures of *C. reinhardtii* exposed to the different concentrations of AMX and in the control cultures without antibiotic. No significant differences in the biomass of cultures were observed during the time of exposure to AMX. ANOVA analysis of the final biomass obtained in the cultures indicated that at all AMX concentrations assayed, including the controls without AMX, and there were no significant differences in both light (*F*_8,18_ = 0.62; *p* = 0.75) and darkness (*F*_8,18_ = 1.88; *p* = 0.13).

**Figure 2:**
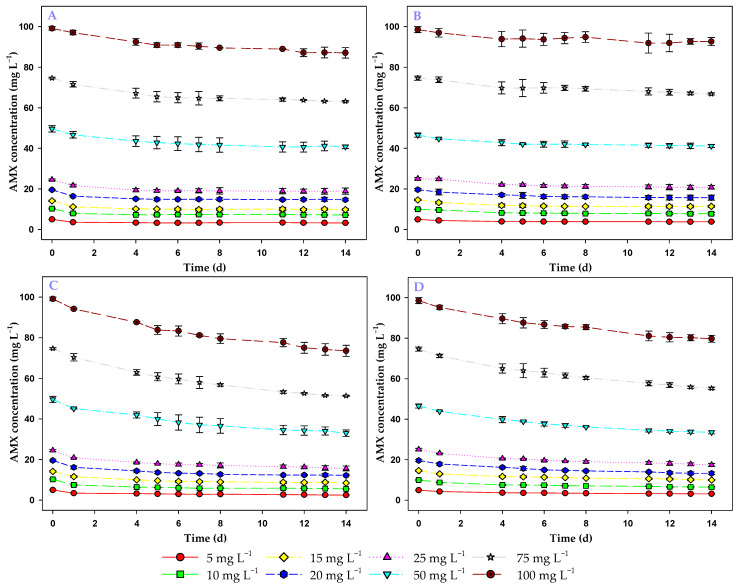
Evolution over time of AMX concentration (mg L^−1^) in the medium using living *C. reinhardtii* biomass, considering biomass alone in light (**A**), biomass alone in darkness (**B**), biomass + abiotic degradation in light (**C**) and biomass + abiotic degradation in darkness (**D**).

Figure 2A,B shows the evolution of the amount of AMX in the medium during the 14 days considering only the effect of the living biomass in the presence of light and in the darkness, respectively. As can be seen in these figures, there was a gradual decrease in the AMX concentration in the medium due to the action of the living biomass of the microalga in both light and darkness. A *t*-test showed that this decrease was significant in the presence of light (*t*_46_ = −5.15; *p* < 0.001) and in darkness (*t*_46_ = −5.21; *p* < 0.001). Therefore, the living biomass of the microalga was able to remove AMX from the medium in both light and darkness. 

If the combined effect of the biomass and abiotic degradation is considered (total process), there was a higher decrease in the amount of AMX present in the medium (Figure 2C,D) compared to the effect of biomass alone. It is important to note that the value attributable to abiotic degradation was lower than the biomass effect when the biomass was exposed to light. However, in darkness the opposite happened; the amount removed by the biomass was less than the amount lost by degradation. Therefore, the living biomass of the microalga in light was the one that contributed most to the removal of AMX during the assay.

At the lowest initial AMX concentration tested (5 mg L^−1^), the living biomass reduced the AMX concentration in the medium to 3.23 ± 0.10 mg L^−1^ in light and 3.80 ± 0.09 mg L^−1^ in darkness. That is, in the presence of light, the living biomass was more effective. For the same concentration, but considering the total process, the AMX concentration in the medium was further reduced, reaching a residual concentration of 2.47 ± 0.01 mg L^−1^ in light and 3.16 ± 0.01 mg L^−1^ in darkness. At the highest AMX concentration tested (100 mg L^−1^), the living biomass was also able to reduce the antibiotic concentration to 88.55 ± 2.59 mg L^−1^ in light and 92.63 ± 1.98 mg L^−1^ in darkness. In the presence of light, the living biomass was also more efficient. Considering the total process, for the same initial AMX concentration, the final AMX concentration in the medium after the assay was 74.97 ± 2.76 mg L^−1^ in light and 79.65 ± 1.69 mg L^−1^ in darkness.

Given these results, it is important to compare the effectiveness of the living biomass in the presence of light versus darkness and if the difference is statistically significant. The analysis of the final concentrations of AMX in the medium in both conditions by means of a *t*-test showed significant differences between light and darkness (*t*_46_ =2.61; *p* = 0.012). At all AMX concentrations tested, the residual amount of the antibiotic was lower in the presence of light than in darkness.

These results show that the living biomass of the microalga *C. reinhardtii* was able to effectively remove AMX from the medium in both light and darkness; however, in the presence of light the efficiency of the living biomass was superior.

### 3.3. Effect of Contact Time and Initial Antibiotic Concentration on the Biosorption Process

**Figure 3:**
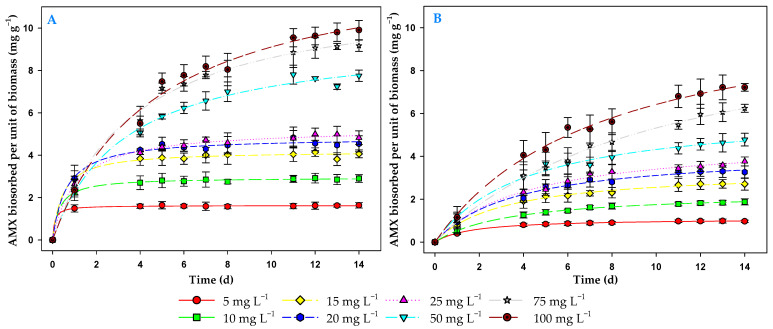
Evolution over time of the amount of AMX biosorbed per unit of biomass (mg g^−1^) in light (**A**) and in darkness (**B**).

To determine the efficiency of the biosorption process, it is useful to use the contact time required to reach a given removal value when equilibrium is reached. Figure 3 illustrates the evolution of the amount of AMX removed per unit of biomass. In this figure it can be seen that the amount of AMX per unit of biomass increased over time until equilibrium was reached at all concentrations tested. At the lowest antibiotic concentrations, equilibrium could be reached in about two days. In contrast, at the highest concentration tested (100 mg L^−1^), equilibrium was reached in about 12 days. Therefore, a 14-day assay was sufficient time to characterize the biosorption process at all tested AMX concentrations. 

**Figure 4:**
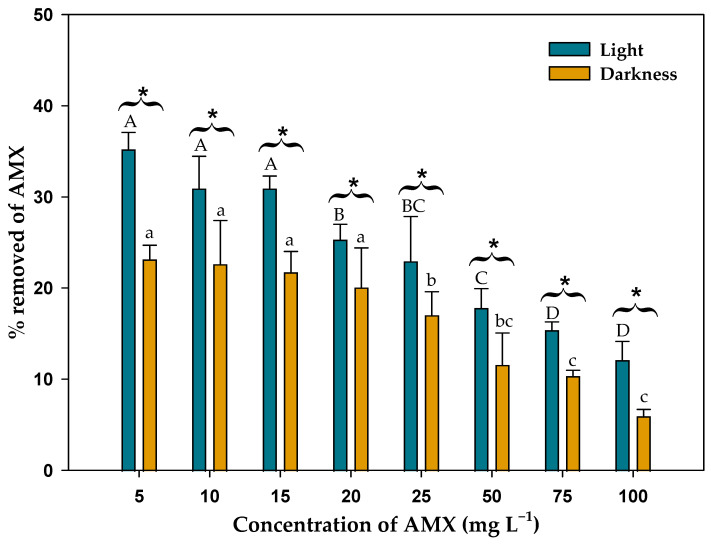
Percentage removed of AMX by the biomass of *C. reinhardtii* in light and darkness after 14 days. Different letters denote significant differences (*p* < 0.05) between the different initial concentrations of AMX in light (upper case letters) and in darkness (lower case letters). * Indicates significant difference (*p* < 0.05) between light and darkness.

Figure 4 shows the percentage of AMX removed by the microalgal biomass as a function of the initial AMX concentration added to the medium after 14 days, both in the presence of light and in the darkness. Analysis of these percentages using a two-way ANOVA, considering light and darkness, and initial AMX concentration as factors, indicated that there were significant differences in the percentage of AMX removed as a function of whether the biomass was exposed to light or in darkness (*F*_1,32_ = 82.51; *p* < 0.001), and also as a function of the initial AMX concentration (*F*_7,32_ = 42.01; *p* < 0.001). Therefore, as with the final AMX concentration in the medium, the percentage removal of AMX by the living biomass was higher in the presence of light compared to darkness. Moreover, this percentage varied significantly as a function of the initial AMX concentration. As the initial AMX concentration increased, the percentage removed decreased. The maximum percentage of AMX removed by the biomass in the presence of light occurred at the 5 mg L^−1^ concentration with 35.2 ± 1.9%, and reached the lowest value at the highest concentration tested (100 mg L^−1^) with 12.1 ± 2.0%. In darkness, these percentages were lower with 23.2 ± 1.5% at the 5 mg L^−1^ concentration, and 6.0 ± 0.7% at the 100 mg L^−1^ concentration.

If the total process is considered, that is, considering the effect of biomass + abiotic degradation, the percentage of AMX eliminated was higher. Thus, in the concentration of 5 mg L^−1^ in the presence of light, the percentage eliminated was 50.4 ± 0.8%, and in darkness, 36.2 ± 0.4%. This difference was due only to the effect of light on the living biomass, and not to its effect on AMX degradation (photodegradation). At the highest AMX concentration (100 mg L^−1^), the effect was similar, but decreasing the percentage removed. Thus, considering the total process, the percentage of AMX removed at this initial AMX concentration was 25.8 ± 2.7% in light, and 19.1 ± 1.9% in darkness.

### 3.4. Kinetic Models

Table 2 and Table 3 show the parameters derived from fitting the data to kinetic models, considering the total process (Table 2), and considering only the effect of biomass (Table 3), in both cases in the presence of light. Three kinetic models, pseudo-first order, pseudo-second order and pseudo-third order were evaluated. For the total process (abiotic degradation + biomass), the best fit was obtained using a pseudo-third order kinetic model, while for the process considering biomass alone, the best fit was with a pseudo-second order kinetic model for all AMX concentrations tested. Considering these results, the total removal process of AMX by the living biomass of the microalga was a complex process resulting from the action of two different processes: (1) the abiotic degradation of AMX that followed a pseudo-first order kinetics, and (2) the biosorption of the antibiotic by the biomass that followed a pseudo-second order kinetics. Therefore, it could be considered that the kinetics of the total process would follow a pseudo-third order kinetics. The good fit of the data to this model (Table 2 and Table 4) allowed for validation of this hypothesis.

Similar kinetic models were obtained for both processes, but in darkness, total process (Table 4) and only biomass effect (Table 5).

The data reflected higher values in the kinetic parameters for light compared to darkness. In fact, the values were higher for both pseudo-third order kinetics for the total process and pseudo-second order kinetics for biomass alone. Equilibrium values (*q_e_*) were higher in the presence of light, indicating a higher amount of AMX removed. Also, the kinetic constants (*k*_2_ and *k*_3_) were higher, indicating a higher removal rate in light than in darkness.

### 3.5. Biosorption Isotherms

**Figure 5:**
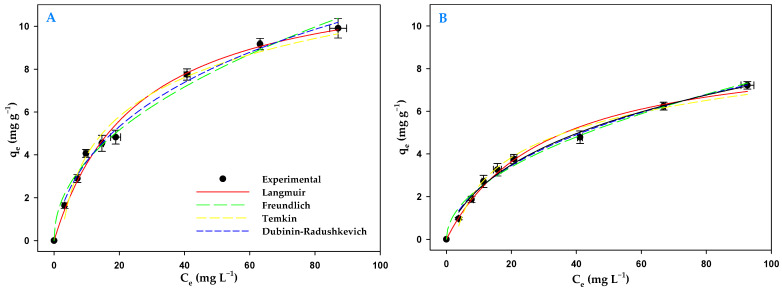
Equilibrium isotherm of AMX biosorption by microalgal biomass in light (**A**) and in darkness (**B**).

It is important to determine the characteristics of a sorbent, such as its sorption capacity, in order to assess its practical use. Biosorption isotherms can be used for this purpose. Figure 5 shows the equilibrium data, and the fits to the different isotherm models, obtained for AMX removal by living cells of *C. reinhardtii* in light and in darkness. The parameters of these models, for both light and darkness, are given in Table 6. Taking into account the *r_adj_*^2^ values obtained, the order of best to worst fit in light was Langmuir > Dubinin–Radushkevich > Freundlich > Temkin. The same order of fit was obtained in darkness. 

Analyzing the data derived from the Langmuir isotherm, the maximum biosorption capacity (*q_max_*) for AMX in light was 12.72 ± 0.57 mg g^−1^, while in darkness it was only 9.25 ± 0.48 mg g^−1^. The *K_F_* parameter of the Freundlich isotherm showed that the living biomass had a higher affinity for AMX in the presence of light with a value of 1.24 ± 0.14 mg L^−1^ while in darkness it was of 0.75 ± 0.08 mg g^−1^. Furthermore, under both conditions, the values obtained for the constant 1/*n* indicated a very favorable biosorption process. With respect to the Temkin isotherm, the *q_t_* constant was also higher in light than in darkness, showing differences between both conditions. The Dubinin–Radushkevich isotherm also provided some insights into the biosorption mechanism. Thus, the constant *E_D_*, which indicates the biosorption free energy, was 8.51 KJ mol^−1^ for light and 8.22 KJ mol^−1^ for darkness, indicating that the process was mainly carried out by chemisorption in both conditions. Although these values fall within chemosorption, they are low values which leads to us considering the process as an ion exchange.

### 3.6. Biomass Characterization

#### 3.6.1. Determination of pH_zcp_

**Figure 6:**
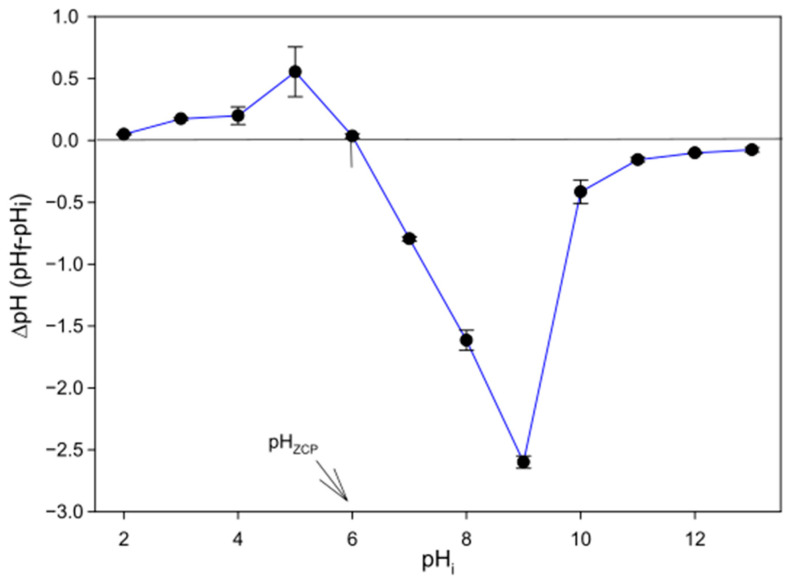
Determination of the zero-charge point (pH_zcp_) of the *C. reinhardtii* biomass.

Figure 6 illustrates the result of the evaluation of the zero-charge point (pH_zcp_) of the biomass of *C. reinhardtii* in freshwater. This point corresponds to the pH at which no variation was obtained between the initial and final pH values. The value obtained for the pH_zcp_ of this biomass was 6.04 ± 0.04.

#### 3.6.2. FTIR

**Figure 7:**
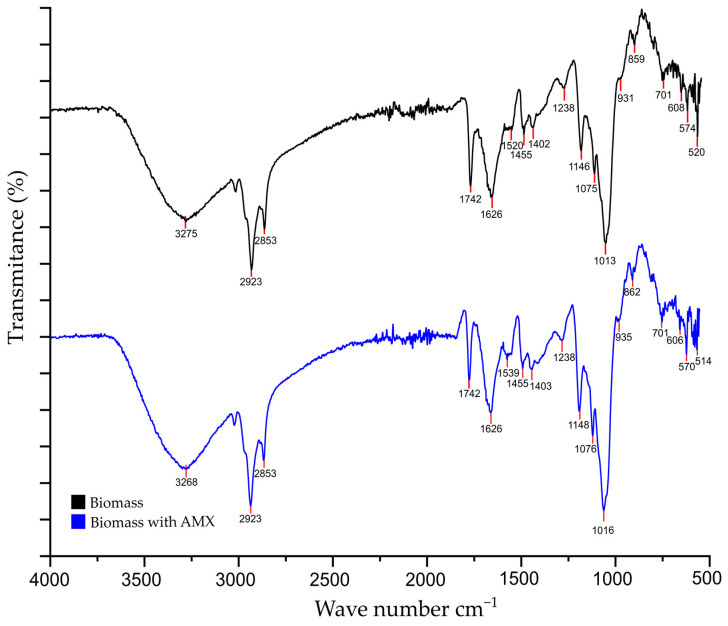
FTIR spectra of *C. reinhardtii* biomass before (black) and after (blue) the AMX biosorption process.

Figure 7 shows the FTIR spectra obtained for the *C. reinhardtii* biomass, considering the biomass alone, and the biomass after a biosorption experiment with AMX. Different characteristic functional groups can be found in the spectrum of the biomass alone. The peak around 3275 cm^−1^ corresponds to the stretching vibrations of –OH [42]. The bands at 2923–2853 cm^−1^ indicate the ion exchange between symmetric or asymmetric C-H protons, and the symmetric CH_2_ stretching vibration of aliphatic acids [43]. The 1742 cm^−1^ band corresponds to the C=O stretching vibrations of carbonyl groups in esters [44]. The 1626–1540 cm^−1^ peaks correspond to C=O stretching of amide group I, belonging mainly to α-helix proteins; while the 1540 cm^−1^ peak is more attributed to amide group II resulting from N-H bending and C-N stretching in proteins [42]. The peak 1455 cm^−1^ corresponds to CH_3_ and CH_2_ bending of methyl groups [45]. The peaks at 1238, 1146 and 1013 cm^−1^ correspond to the P=O and C-N bending of amide III of proteins, phospholipids and carbohydrates associated with nucleic acids, and stretching of C-O-C of polysaccharides [42], respectively.

Comparing the wavenumbers of the characteristic peaks of the biomass, such as 3275, 2923, 1520, 1238, 1146 and 1013 cm^−1^ with those after AMX biosorption, there was a shift and/or increase at 3268, 2923, 1539, 1238, 1148 and 1016 cm^−1^. The band around 3275–3268 cm^−1^ was more prominent due to the -OH present in AMX, which could interact with the biomass through hydrogen bond formation [46]. The peak at 2923 cm^−1^ also increased after biosorption, which could be due to the -COOH vibrations of the carboxyl groups present in AMX [47]. The observed shift in peaks 1539, 1148 and 1016 cm^−1^ could be due to the interaction with AMX. The increase in the peak at 1238 cm^−1^ can be associated with the C=C stretching of the benzene ring of AMX [48]. These results confirm that AMX interacted with the living biomass of the microalga during the biosorption process.

### 3.7. Effect of pH on Biosorption Efficiency

First, the stability of AMX at the different pHs tested was evaluated using controls without microalgal biomass. After 14 days of testing, there was a reduction in the initial concentration of AMX at all concentrations tested and at all pHs tested. The residual concentration of the antibiotic obtained in these experiments were analyzed using a three-way ANOVA considering the factors of the initial and final time, the initial concentration of the antibiotic and the pH value. This analysis showed that there were significant differences in the three factors, that is, in the AMX concentration as a function of time (*F*_1,60_ = 1416.15; *p* < 0.001), in initial AMX concentration (*F*_2,60_ = 62,177.49; *p* < 0.001) and in the effect of pH (*F*_3,60_ = 11.29; *p* < 0.001). These data indicated that AMX had significant abiotic degradation throughout the 14 days at all concentrations (as expected and demonstrated above), but with the effect of pH being more relevant in more alkaline media. Thus, the effect of pH 10 was significantly different from the other pHs tested, increasing the abiotic degradation of AMX.

**Figure 8:**
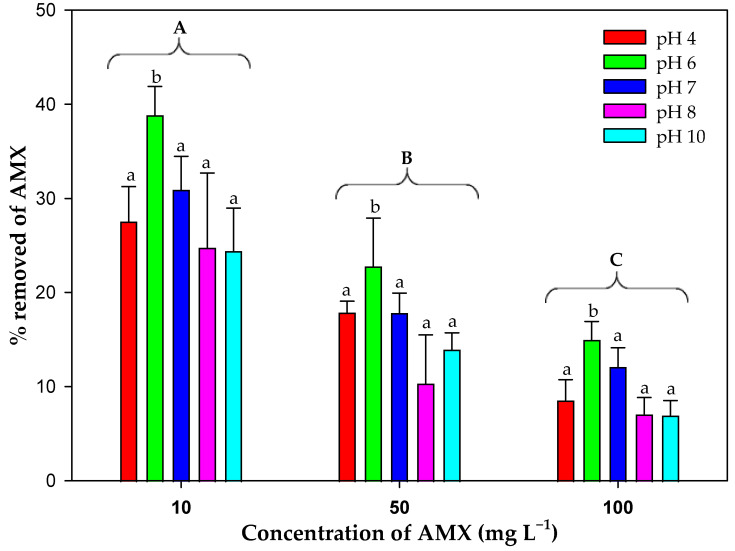
Percentage of AMX removed by microalgal biomass after 14 days depending on the pH and initial concentration. Different letters denote significant differences (*p* < 0.05) in the percentage of AMX removal at the different pHs tested (lower case), and between the initial AMX concentrations (upper case).

Figure 8 shows the percentage of antibiotic removed by the microalgal biomass at the different pHs tested without considering abiotic degradation. The results analyzed by a three-way ANOVA (factors: initial and final time, initial antibiotic concentration tested and pH value) showed that there were significant differences for time (*F*_1,60_ = 1096.86; *p* < 0.001), and for pH value (*F*_3,60_ = 13.29; *p* < 0.001) at all initial AMX concentrations (*F*_2,60_ = 102.17; *p* < 0.001). This analysis confirmed that there was a significant difference in the AMX removed by the living biomass at the different pHs tested. The biomass removed the antibiotic at all pHs tested; however, the pH influenced the removal efficiency. pH 6 showed the highest removal value for the three concentrations tested, with the percentage decreasing significantly as the initial AMX concentration increased. Therefore pH 6 would be the optimal pH value for AMX removal by the living biomass of the microalga.

## 4. Discussion

Today, the presence of pollutants, such as antibiotics, in water bodies is well known and leads to direct and indirect problems in the affected environments [9,10,11,12,13]. Therefore, works such as the present study are important to focus on and look for possible solutions to this problem. Biosorption is considered a very effective technique to remove pollutants in an environmentally friendly way. The search for the most efficient biomass for the removal of a given pollutant is important in order to develop the process properly. Biosorption studies to remove the antibiotic AMX from aqueous solutions are few, which leads to study the capacity of new biomasses to remove this antibiotic effectively. Correctly assessing the efficacy of a given biomass is the key to developing a suitable biosorption process. One issue that should be considered in biosorption is the assessment of the stability of the pollutant under the test conditions. However, verifying the stability of the pollutant (and therefore its possible spontaneous decrease throughout the experiment) under the test conditions prior to the study is uncommon in biosorption studies [49]. This fact would lead to attributing this reduction to the biomass, which would be overestimated in terms of its biosorption capacity. However, many pollutants lose stability when they are solubilized or exposed to certain environmental conditions such as illumination (photodegradation). Since living biomass of microalgae was used in the present work, illumination was necessary to maintain the metabolic characteristics of the cells; however, it can be an important factor in relation to the stability of the pollutant. In the case of AMX, it was possible to verify the spontaneous reduction in this antibiotic over time due to abiotic factors. However, under the illumination conditions of the experiments, photodegradation did not contribute to increasing the reduction in this antibiotic. This result agrees with other studies that confirmed that in the presence of light (for example, irradiation by sunlight) there was no significant photodegradation of AMX [50,51]. This degradation by abiotic factors is mainly produced by hydrolysis, which affects the amino side chains on the carbonyl present in β-lactam compounds [52], as in the case of AMX.

Based on the results obtained, it was possible to determine the good capacity of the living biomass of the microalga *C. reinhardtii* to remove AMX from an aqueous medium. Despite the spontaneous reduction in the antibiotic, the presence of the microalgal biomass significantly increased the removal of this antibiotic, both in quantity and speed. The amount of AMX removed per unit of biomass at a given time was higher than the amount lost by spontaneous degradation. In fact, microalgae are considered among the so-called emerging biosorbents, as they have been shown to be very effective in the removal of pollutants [53], including antibiotics [54]. Green algae present various components in their cell wall that provide binding sites such as hydroxyl, carboxyl, amino and sulfhydryl [55,56], where these microalgal surface features facilitate the capture of pollutants, and constitute an important role in the biosorption process. Furthermore, the type of biomass used (living or dead) can influence the efficiency of the biosorption process [28,57]. Therefore, it is important to assess the effect of living biomass in this type of process.

Figure 4 is a good indicator of the efficiency of the living biomass of this microalga to remove AMX from the medium. The biomass of this microalga was able to remove 35.2 ± 1.9% of AMX when the initial concentration of this antibiotic was 5 mg L^−1^. Although this was the lowest concentration used in the experiments (necessary to properly perform biosorption experiments), this concentration can be considered high compared to levels found in natural environments. For example, values around of 1 ng L^−1^ can be found in surface water [58] and of 1.80–120 ng L^−1^ in wastewater effluents [59]. Considering these values and the results obtained in the experiments, the efficiency of this microalga may be much higher with percentages that could be considered close to 100%. However, it is also important to consider the different efficiency of the microalga in the presence of light or in darkness. The efficiency of the biomass in the presence of light was always higher than the efficiency in darkness. Even in darkness, biomass efficiency was lower than the amount of AMX lost to abiotic degradation. The kinetic equations (Table 2, Table 3, Table 4 and Table 5) also supported this result, the biosorption rates were also higher when the biomass was exposed to light than in darkness. This difference in light and darkness is closely related to the photosynthetic capabilities of the microalgae and their bioactivity upon exposure to a light source. There is a good correlation between photochemical yield and biomass under light [60]. In general, adsorption to the cell surface of biomass is a common mechanism of pollutant removal; however, in the presence of light, the living microalgae have a higher metabolic activity, facilitating that AMX can also be removed by bioaccumulation inside the cell or even degraded by cell metabolism. It is well known that increasing the intensity of light stimulates nutrient uptake by microalgae [61]. Thus, nutrients are incorporated in greater quantities into the cell interior due to the greater activity of the transport systems that require energy for their function. This mechanism could be extended to other compounds, such as AMX. This antibiotic could be incorporated into the microalgal cells by active transport, facilitating the incorporation of this antibiotic into the cell interior, which would explain that in the presence of light, the removal efficiency would be higher. In darkness, metabolic activity would be more reduced, and therefore, the activity of the active transport systems, so that removal would be mostly by adsorption to the surface or by diffusion. Therefore, the use of living biomass under optimal conditions is superior to the use of dead biomass.

The data obtained from the equilibrium isotherms (Table 6, Figure 5) for the biosorption process by the biomass provided information on this process. Thus, the Langmuir isotherm gave the maximum biosorption capacity of AMX by this biomass (12.72 ± 0.57 mg L^−1^). This value was also higher in the light than in darkness. The parameters derived from the Freundlich isotherm model also provided information on the properties of the biomass. The *K_F_* parameter indicated a higher affinity of *C. reinhardtii* biomass for AMX in the light than in the darkness. A similar situation occurred with the 1/*n* parameter of this model, which is related to the intensity of sorption and the heterogeneity of the material. The values obtained for this biomass were less than one, indicating favorable sorption with heterogeneous material. Taking this parameter into account, biosorption was also more favorable in the presence of light. Both in light and darkness, the values of the *Bt* constant of the Temkin isotherm model were positive, indicating that the biosorption of AMX by the living biomass of this microalga was an exothermic process [62]. Finally, the Dubinin–Radushkevich model also indicated that the energy value of AMX biosorption was higher in light than in darkness. The values obtained for this energy were slightly higher than 8 KJ mol^−1^, indicating that chemisorption may have been involved in the biosorption process [63]. However, the energy values obtained were very close to the borderline between physisorption and chemisorption. For this reason, considering ion exchange as a main mechanism would be more reasonable. In spite of this, covalent binding of AMX to functional groups of the microalgal biomass is feasible, as indicated by the FTIR spectrum, and the use of living biomass may be the main reason for this high biosorption energy. AMX can enter the cell interior requiring additional energy compared to the adsorption to the cell surface that would occur using dead biomass. In fact, the process had a higher energy value in light than in darkness.

For biosorption studies it is of vital importance to determine the influence of factors such as pH, because it has a direct effect on the pollutant removal since the pH of the medium can vary the electrostatic forces varying the maximum capacity of the biomasses, in this case, the capacity of the microalga *C. reinhardtii*. Considering the instability detected in AMX, it is important to know whether pH also influenced this instability. The results obtained indicated that this instability increased at alkaline pHs. This result is consistent with other studies that indicated that AMX is a more stable compound at low pH [64], and losing stability at alkaline pH [52] due mainly to the deprotonation of the amine group of this molecule [65]. Figure 8 shows the removal capacity of *C. reinhardtii* for the different pHs tested, indicating that the highest efficiency was obtained at pH 6 (at this pH there was no significant increase in abiotic degradation). The explanation for this result is related to the charges present both on the surface of the biomass and in the AMX molecule. The charges of the biomass depend on the value of the zero-charge point (pH_zcp_) of this biomass. The value obtained was 6.04 ± 0.04 (Figure 6). The biomass would be positively charged at pHs below this value and negatively charged above it. Regarding the charge of AMX, the pK_a_ of this compound must be taken into account. In this case, AMX has three pK_a_, 2.7, 7.5 and 9.6 [66,67]. The isoelectric point would be located at around pH 5. In general, the charges of the AMX molecule are more negative as the pH of the medium increases from that value and more positive as the pH of the medium decreases. This behavior is a consequence of the carboxyl and amino functional groups of AMX that confer amphoteric properties, so that at high pH, the carboxyl groups dissociate creating net negative charges on the molecule. However, at low pH, the amino groups absorb hydrogen atoms, which results in a net positive charge [66]. Considering the values of pK_a_ of AMX and the pH_zcp_ of the biomass, at pH values below 2.7, both AMX and biomass would be positively charged generating repulsion, and therefore lower removal capacity. As the pH rises above 2.7, the charge of the AMX becomes more neutral as in the biomass and this repulsive force weakens, until a pH 6 is reached, which coincides with the point of zero charge of the living biomass, reaching the maximum removal capacity of the biomass at this pH. As the pH continues to increase, above pH 6 this repulsion strengthens again because the negative charge of AMX and of the living biomass increases, reducing the biosorption capacity.

Table 7 summarizes the results obtained on the efficiency (%) of AMX removal by the living biomass of this microalga as a function of the factors tested. It can be observed that light and pH = 6 are the optimal conditions for higher efficiency when this biomass is used.

For a better understanding of the interaction of AMX with the microalgal biomass, a FTIR analysis was carried out and whose spectrum (Figure 7) showed the presence of functional groups in the microalgal biomass with which AMX could interact and their influence on AMX uptake. This analysis suggests an interaction of AMX with hydroxyl, carboxyl and amino groups in the biosorption process [68] through a hydrophilic interaction [56] which reinforces the idea that ion exchange would be one of the main mechanisms of interaction between AMX and this biomass.

For biosorption studies, it is important to highlight which materials or organisms have the best efficiency for the removal of a given pollutant. For this purpose, it is necessary to be able to compare the results obtained with other sorbents. Thus, Table 8 could serve as a reference to compare the different values obtained in AMX removal investigations using different materials.

As can be seen in Table 8, the studies carried out for AMX removal use materials of different nature. Typical adsorbents, such as activated carbon, showed very favorable results; however, these techniques are subject to very laborious elaboration with significant emissions to the environment and are difficult to transfer to the natural environment. If the results obtained with the microalgal biomass are compared with other microorganisms, the biomass of *C. reinhardtii* had a higher affinity for AMX and a higher value of the pseudo-second order kinetic constant at the concentrations tested, indicating a higher rate of AMX uptake from the medium. These properties result in a biomass capable of showing good affinity and having a high rate of uptake of AMX from the medium, demonstrating that this biomass is capable of efficiently removing AMX. In addition, the use of this biomass is an easy method to use, with the possibility of transferring it to the natural environment and a low-cost technique.

## 5. Conclusions

It is important to check the stability of a pollutant in biosorption studies beforehand in order to correctly assess the properties of a biomass. The living biomass of the microalga *Chlamydomonas reinhardtii* has shown good biosorption characteristics to remove AMX in polluted freshwater. The living biomass of this microalga had a maximum AMX biosorption capacity of 12.72 ± 0.57 mg g^−1^ in the presence of light which meant an efficiency of 12.1 ± 2.0% at a high initial AMX concentration (100 mg L^−1^). Lighting was an important factor in increasing the removal capacity of this biomass. The removal of AMX by this biomass followed a pseudo-second-order kinetics with *k_2_* values of 0.02–6.79 (g mg^−1^ h^−1^), and a pH of 6 in the medium was the optimum to obtain a higher efficiency. The use of living biomass under optimal conditions would be more suitable for the removal of this pollutant than the use of dead biomass.

## Figures and Tables

**Table 1:** Equations of the kinetic and isotherm models used in this study.

Kinetics	Isotherms
Pseudo-first order [33]	Langmuir [34]
$q=q_{e}(1-e^{-k_{1}t})$ (3)	$q_{e}=\frac{\left(q_{max}K_{L}C_{e}\right)}{\left(1+K_{L}C_{e}\right)}$ (6)
Pseudo-second order [35]	Freundlich [36]
$q=\frac{q_{e}^{2}k_{2}t}{1+q_{e}k_{2}t}$ (4)	qe=KFCe1n (7)
Pseudo-third order [37,38]	Temkin [39,40]
$q=q_{e}-(\frac{q_{e}}{\sqrt{2{q_{e}}^{2}k_{3}t+1}})$ (5)	$q_{e}=q_{T}\times ln(A_{T}C_{e})$ (8)
	Dubinin–Radushkevich [41]
	$q_{e}=q_{max}\times e^{-B_{D}{\left(RT\times ln\left(\left(\frac{sol}{C_{e}}\right)\right)\right)}^{2}}$ (9) $E_{D}=\frac{1}{\sqrt[{2}]{2B_{D}}}$ (10)
*q* (mg g^−1^) is the mass of AMX biosorbed per unit of biomass over time *t* (d), *q_e_* (mg g^−1^) is the mass of AMX biosorbed per unit of biomass at equilibrium, *k*_1_ (d^−1^) is the constant of the pseudo-first order kinetic model, *k*_2_ (g mg^−1^ d^−1^) is the constant of the pseudo-second order kinetic model and *k*_3_ (g^2^ mg^−2^ d^−1^) is the constant of the pseudo-third order kinetic model.	*q_e_* (mg g^−1^) is the mass of AMX biosorbed at equilibrium per unit of biomass, *q_max_* (mg g^−1^) is the maximum sorption capacity, *q_T_* (mg g^−1^) is the mass of AMX biosorbed per unit of mass over time, *K_L_* (L mg^−1^) is the affinity constant of the material, *C_e_* (mg L^−1^) is the concentration of AMX at equilibrium, *K_F_* (L mg^−1^) is the Freundlich constant, *n* the intensity of the sorption, *A_T_* (L g^−1^) is the binding energy constant, *R* is the gas constant (0.008314 KJ mol^−1^ K^−1^), *T* is temperature at 291 K, *B_D_* is the free energy of sorption per mole of sorbate (mol^2^ KJ^−2^), *E_D_* (KJ mol^−1^) is the apparent energy of biosorption and *sol* is the solubility of the antibiotic (mg/L).

**Table 2:** Parameters of the kinetic models for AMX removal by living microalgal biomass considering the total process (biomass + abiotic degradation) in light.

Initial Concentration (mg L^−1^)	Pseudo-First Order			Pseudo-Second Order			Pseudo-Third Order		
	*q_e_* (mg L^−1^)	*k*_1_ (h^−1^)	*r_adj_* ^2^	*q_e_* (mg L^−1^)	*k*_2_ (L mg^−1^ h^−1^)	*r_adj_* ^2^	*q_e_* (mg L^−1^)	*k*_3_ (L^2^ mg^−2^ d^−1^)	*r_adj_* ^2^
5	2.18 ± 0.08	1.16 ± 0.36	0.8817	2.42 ± 0.11	0.50 ± 0.19	0.9367	2.75 ± 0.15	0.19 ± 0.09	0.9536
10	4.38 ± 0.08	0.95 ± 0.14	0.9702	4.80 ± 0.07	0.27 ± 0.03	0.9930	5.41 ± 0.09	0.05 ± 8 × 10^−7^	0.9958
15	5.36 ± 0.13	0.45 ± 0.06	0.9730	6.12 ± 0.12	0.10 ± 0.01	0.9935	7.17 ± 0.15	0.01 ± 2 × 10^−3^	0.9953
20	7.11 ± 0.22	0.40 ± 0.06	0.9612	8.12 ± 0.23	0.07 ± 0.01	0.9870	9.55 ± 0.29	7 × 10^−3^ ± 1 × 10^−3^	0.9916
25	8.44 ± 0.33	0.32 ± 0.05	0.9566	9.87 ± 0.38	0.04 ± 8 × 10^−3^	0.9829	11.78 ± 0.45	3 × 10^−3^ ± 7 × 10^−4^	0.9894
50	18.15 ± 1.00	0.16 ± 0.02	0.9806	24.93 ± 1.96	5 × 10^−3^ ± 1 × 10^−3^	0.9828	32.13 ± 2.87	1 × 10^−4^ ± 4 × 10^−5^	0.9836
75	27.19 ± 0.65	0.14 ± 7 × 10^−3^	0.9976	38.87 ± 1.00	3 × 10^−3^ ± 2 × 10^−4^	0.9987	51.18 ± 1.37	3 × 10^−5^ ± 4 × 10^−6^	0.9989
100	30.11 ± 1.52	0.13 ± 0.01	0.9909	43.61 ± 2.92	2 × 10^−3^ ± 4 × 10^−4^	0.9922	57.74 ± 4.30	2 × 10^−5^ ± 7 × 10^−6^	0.9926

**Table 3:** Parameters of the kinetic models for AMX removal considering only the effect of the microalgal biomass in light.

Initial Concentration (mg L^−1^)	Pseudo-First Order			Pseudo-Second Order			Pseudo-Third Order		
	*q_e_* (mg g^−1^)	*k*_1_ (h^−1^)	*r_adj_* ^2^	*q_e_* (mg g^−1^)	*k*_2_ (g mg^−1^ h^−1^)	*r_adj_* ^2^	*q_e_* (mg g^−1^)	*k*_3_ (g^2^ mg^−2^ d^−1^)	*r_adj_* ^2^
5	1.61 ± 0.01	2.61 ± 0.19	0.9981	1.63 ± 0.01	6.79 ± 1.37	0.9982	1.67 ± 0.02	17.07 ± 6.76	0.9980
10	2.83 ± 0.02	1.57 ± 0.11	0.9937	2.95 ± 0.02	1.07 ± 0.10	0.9978	3.17 ± 0.03	0.57 ± 0.09	0.9976
15	3.97 ± 0.04	1.27 ± 0.10	0.9917	4.19 ± 0.05	0.53 ± 0.07	0.9934	4.56 ± 0.09	0.17 ± 0.05	0.9905
20	4.49 ± 0.05	1.02 ± 0.10	0.9881	4.84 ± 0.08	0.33 ± 0.05	0.9899	5.38 ± 0.15	0.07 ± 0.02	0.9859
25	4.73 ± 0.08	0.69 ± 0.09	0.9789	5.29 ± 0.06	0.18 ± 0.02	0.9965	6.06 ± 0.10	0.03 ± 4 × 10^−3^	0.9963
50	7.82 ± 0.16	0.28 ± 0.02	0.9910	9.63 ± 0.32	0.03 ± 4 × 10^−3^	0.9919	11.81 ± 0.52	2 × 10^−3^ ± 5 × 10^−4^	0.9908
75	9.42 ± 0.17	0.26 ± 0.02	0.9941	11.78 ± 0.34	0.02 ± 2 × 10^−3^	0.9946	14.58 ± 0.57	1 × 10^−3^ ± 2 × 10^−4^	0.9935
100	10.34 ± 0.31	0.22 ± 0.02	0.9888	13.39 ± 0.65	0.02 ± 2 × 10^−3^	0.9889	16.84 ± 1.03	7 × 10^−4^ ± 2 × 10^−4^	0.9881

**Table 4:** Parameters of the kinetic models for AMX removal by living microalgal biomass considering the total process (biomass + abiotic degradation) in darkness.

Initial Concentration (mg L^−1^)	Pseudo-First Order			Pseudo-Second Order			Pseudo-Third Order		
	*q_e_* (mg L^−1^)	*k*_1_ (h^−1^)	*r_adj_* ^2^	*q_e_* (mg L^−1^)	*k*_2_ (L mg^−1^ h^−1^)	*r_adj_* ^2^	*q_e_* (mg L^−1^)	*k*_3_ (L^2^ mg^−2^ d^−1^)	*r_adj_* ^2^
5	1.74 ± 0.06	0.31 ± 0.04	0.9702	2.06 ± 0.06	0.19 ± 0.03	0.9907	2.47 ± 0.07	0.06 ± 0.01	0.9951
10	3.46 ± 0.14	0.27 ± 0.04	0.9683	4.19 ± 0.18	0.08 ± 0.01	0.9858	5.09 ± 0.22	0.01 ± 2 × 10^−3^	0.9901
15	4.45 ± 0.21	0.24 ± 0.04	0.9609	5.45 ± 0.30	0.05 ± 0.01	0.9788	6.65 ± 0.39	6 × 10^−3^ ± 2 × 10^−3^	0.9842
20	6.78 ± 0.27	0.19 ± 0.02	0.9857	8.98 ± 0.47	0.02 ± 3 × 10^−3^	0.9898	11.38 ± 0.66	1 × 10^−3^ ± 3 × 10^−4^	0.9911
25	7.70 ± 0.25	0.20 ± 0.02	0.9886	10.05 ± 0.38	0.02 ± 3 × 10^−3^	0.9940	12.67 ± 0.51	1 × 10^−3^ ± 2 × 10^−4^	0.9952
50	14.77 ± 0.32	0.15 ± 7 × 10^−3^	0.9974	20.67 ± 0.57	6 × 10^−3^ ± 5 × 10^−4^	0.9982	26.95 ± 0.82	1 × 10^−4^ ± 2 × 10^−5^	0.9984
75	23.25 ± 0.88	0.12 ± 9 × 10^−3^	0.9957	34.06 ± 1.42	3 × 10^−3^ ± 3 × 10^−4^	0.9974	45.33 ± 1.94	4 × 10^−5^ ± 6 × 10^−6^	0.9978
100	23.46 ± 1.28	0.12 ± 0.01	0.9919	34.66 ± 2.42	3 × 10^−3^ ± 5 × 10^−4^	0.9932	46.29 ± 3.54	3 × 10^−5^ ± 9 × 10^−6^	0.9935

**Table 5:** Parameters of the kinetic models for AMX removal considering only the effect of the microalgal biomass in darkness.

Initial Concentration (mg L^−1^)	Pseudo-First Order			Pseudo-Second Order			Pseudo-Third Order		
	*q_e_* (mg g^−1^)	*k*_1_ (h^−1^)	*r_adj_* ^2^	*q_e_* (mg g^−1^)	*k*_2_ (g mg^−1^ h^−1^)	*r_adj_* ^2^	*q_e_* (mg g^−1^)	*k*_3_ (g^2^ mg^−2^ d^−1^)	*r_adj_* ^2^
5	0.96 ± 0.01	0.45 ± 0.03	0.9901	1.11 ± 0.01	0.54 ± 0.04	0.9980	1.31 ± 0.03	0.37 ± 0.05	0.9956
10	1.89 ± 0.02	0.27 ± 0.01	0.9976	2.35 ± 0.04	0.13 ± 8 × 10^−3^	0.9984	2.89 ± 0.07	0.03 ± 4 × 10^−3^	0.9970
15	2.74 ± 0.06	0.29 ± 0.02	0.9900	3.35 ± 0.08	0.10 ± 0.01	0.9958	4.10 ± 0.12	0.02 ± 3 × 10^−3^	0.9955
20	3.39 ± 0.08	0.26 ± 0.02	0.9904	4.23 ± 0.16	0.07 ± 0.01	0.9909	5.22 ± 0.25	0.01 ± 2 × 10^−3^	0.9898
25	3.79 ± 0.09	0.24 ± 0.02	0.9917	4.82 ± 0.18	0.05 ± 7 × 10^−3^	0.9927	6.00 ± 0.28	6 × 10^−3^ ± 1 × 10^−3^	0.9921
50	4.80 ± 0.13	0.24 ± 0.02	0.9886	6.13 ± 0.26	0.04 ± 7 × 10^−3^	0.9904	7.67 ± 0.42	4 × 10^−3^ ± 1 × 10^−4^	0.9895
75	7.51 ± 0.25	0.13 ± 8 × 10^−3^	0.9963	10.99 ± 0.47	0.01 ± 1 × 10^−3^	0.9975	14.63 ± 0.70	3 × 10^−4^ ± 6 × 10^−5^	0.9974
100	8.12 ± 0.26	0.16 ± 0.01	0.9942	11.30 ± 0.54	0.01 ± 1 × 10^−3^	0.9943	14.73 ± 0.84	3 × 10^−4^ ± 1 × 10^−4^	0.9941

**Table 6:** Isotherm model parameters of AMX biosorption by microalgal biomass in the presence of light and in darkness.

Isotherm	Parameters	Light	Darkness
**Langmuir**	***q_max_*** (mg g^−1^)	12.72 ± 0.57	9.25 ± 0.48
	***K_L_*** (L mg^−1^)	0.04 ± 4 × 10^−3^	0.03 ± 4 × 10^−3^
	***r_adj_* ^2^**	0.9909	0.9896
**Freundlich**	***K_F_*** (L mg^−1^)	1.24 ± 0.14	0.75 ± 0.08
	**1/n**	0.48 ± 0.03	0.50 ± 0.03
	***r_adj_* ^2^**	0.9860	0.9871
**Temkin**	***b_T_*** (J mol^−1^)	2.62 ± 0.17	1.93 ± 0.10
	***A_T_*** (L mg^−1^)	0.46 ± 0.07	0.36 ± 0.04
	***r_adj_* ^2^**	0.9720	0.9798
**Dubinin–** **Radushkevich**	***q_max_*** (mg g^−1^)	25.20 ± 1.52	18.51 ± 1.13
	***B_D_*** (mol^2^ KJ^−2^)	6.9 × 10^−3^ ± 3 × 10^−4^	7.4 × 10^−3^ ± 3 × 10^−4^
	***E_D_*** (KJ mol^−1^)	8.51 ± 0.13	8.22 ± 0.12
	***r_adj_* ^2^**	0.9892	0.9897

**Table 7:** Summary of the AMX removal efficiency (%) by the living biomass of the microalga as a function of the initial concentration of the antibiotic and taking into account the analyzed factors.

	Removed AMX (%)							
Initial AMX Concentration (mg L^−1^)	Light (pH = 7)		Darkness (pH = 7)		pH (Light, Microalgal Biomass)			
	Abiotic Degradation + Biomass	Microalgal Biomass	Abiotic Degradation + Biomass	Microalgal Biomass	4	6	8	10
**5**	50.38 ± 0.79	35.20 ± 1.87	36.21 ± 0.43	23.16 ± 1.54	N.D.	N.D.	N.D.	N.D.
**10**	46.29 ± 1.38	30.92 ± 2.55	35.81 ± 0.85	22.64 ± 3.79	27.56 ± 3.70	38.82 ± 3.07	24.78 ± 2.64	24.42 ± 4.53
**15**	40.22 ± 1.58	30.92 ± 1.38	31.49 ± 0.97	21.74 ± 0.28	N.D.	N.D.	N.D.	N.D.
**20**	37.64 ± 0.52	25.32 ± 0.67	32.32 ± 1.85	20.07 ± 4.34	N.D.	N.D.	N.D.	N.D.
**25**	36.37 ± 3.91	22.96 ± 4.89	30.16 ± 2.71	17.04 ± 2.56	N.D.	N.D.	N.D.	N.D.
**50**	33.43 ± 3.63	17.83 ± 2.11	27.93 ± 1.22	11.61 ± 3.45	17.88 ± 1.20	22.79 ± 5.10	10.35 ± 3.96	13.97 ± 1.74
**75**	31.14 ± 0.48	15.40 ± 0.87	25.87 ± 1.54	10.38 ± 0.60	N.D.	N.D.	N.D.	N.D.
**100**	25.82 ± 2.90	12.13 ± 2.01	19.14 ± 1.91	5.97 ± 0.71	8.56 ± 2.18	14.99 ± 1.93	7.08 ± 1.75	6.98 ± 1.53

N.D.: “Not determined”.

**Table 8:** Characteristics of different sorbents used for AMX removal.

Adsorbents	*q_max_* ^†^ (mg g^−1^)	*K_F_* ^††^ (L mg^−1^)	Pseudo-Second Order Kinetic Constant (g mg^−1^ d^−1^)	Biomass (g L^−1^)	Initial AMX Concentration (mg L^−1^)	References
Activated carbon with induced NH_4_Cl	438.6	76.3	48.0–0.24	0.8	10–100	[19]
Olive biomass	237.0	64.4	2 × 10^−2^–9 × 10^−2^	0.6	400–800	[69]
*Pithophora*	25.8	1.2	1.4–7 × 10^−2^	5	10–150	[46]
*Chlamydomonas reinhardtii*	12.7	1.2	6.8–2 × 10^−2^	1.1	5–100	This work
*Saccharomyces cerevisiae*	6.3	0.5	0.1	5	5–25	[70]

^†^ Parameter obtained from a Langmuir isotherm. ^††^ Freundlich constant.

## Data Availability

The data sets generated during or analyzed during the current study are available from the corresponding author on reasonable request.
